# Structural insight into operator *dre*-sites recognition and effector binding in the GntR/HutC transcription regulator NagR

**DOI:** 10.1093/nar/gku1374

**Published:** 2015-01-06

**Authors:** Simon B. Fillenberg, Florian C. Grau, Gerald Seidel, Yves A. Muller

**Affiliations:** 1 Lehrstuhl für Biotechnik, Department of Biology, Friedrich-Alexander University Erlangen-Nuremberg, Henkestrasse 91, D-91052 Erlangen, Germany; 2 Lehrstuhl für Mikrobiologie, Department of Biology, Friedrich-Alexander University Erlangen-Nuremberg, Staudtstrasse 5, D-91058 Erlangen, Germany

## Abstract

The uptake and metabolism of *N*-acetylglucosamine (GlcNAc) in *Bacillus subtilis* is controlled by NagR (formerly named YvoA), a member of the widely-occurring GntR/HutC family of transcription regulators. Upon binding to specific DNA operator sites (*dre*-sites) NagR blocks the transcription of genes for GlcNAc utilization and interaction of NagR with effectors abrogates gene repression. Here we report crystal structures of NagR in complex with operator DNA and in complex with the putative effector molecules glucosamine-6-phosphate (GlcN-6-P) and *N*-acetylglucosamine-6-phosphate (GlcNAc-6-P). A comparison of the distinct conformational states suggests that effectors are able to displace the NagR–DNA-binding domains (NagR–DBDs) by almost 70 Å upon binding. In addition, a high-resolution crystal structure of isolated NagR–DBDs in complex with palindromic double-stranded DNA (dsDNA) discloses both the determinants for highly sequence-specific operator *dre*-site recognition and for the unspecific binding of NagR to dsDNA. Extensive biochemical binding studies investigating the affinities of full-length NagR and isolated NagR–DBDs for either random DNA, *dre*-site-derived palindromic or naturally occurring non-palindromic *dre*-site sequences suggest that proper NagR function relies on an effector-induced fine-tuning of the DNA-binding affinities of NagR and not on a complete abrogation of its DNA binding.

## INTRODUCTION

Bacteria have evolved elaborate strategies to swiftly adapt to changing carbon sources (1,2). These mechanisms involve, among others, regulator proteins that either repress or activate gene transcription through binding to specific DNA-operator sequences. NagR, formerly termed YvoA, has been identified as a repressor protein from *Bacillus subtilis* that regulates genes involved in *N*-acetylglucosamine (GlcNAc) utilization (3). GlcNAc, a highly valuable carbon-nitrogen source in the absence of glucose, is readily available in the biosphere and serves as a common building block in high-abundant biopolymers, such as chitin, a principal constituent of the exoskeleton of arthropods, chitosan from fungi and peptidoglycan from bacterial cell walls (3–5).

NagR acts as a negative transcriptional regulator, and binding of effector-free NagR to specific DNA operator sites blocks gene transcription. NagR recognizes operator sites highly similar to those of the homologous protein DasR from *Streptomyces coelicolor* (6,7). Therefore these sites are also termed DasR-responsive elements (*dre*-sites) in *B. subtilis* (8). *Dre*-sites occur in *B. subtilis* in the promoter region of the *nagA-nagB-nagR* and *nagP* gene locus (3). *NagP* encodes for a GlcNAc-specific subunit of the sugar phosphotransferase system which is involved in transport and at the same time in phosphorylation of GlcNAc (3,9–10). The gene product of *nagA* is a *N*-acetylglucosamine-6-phosphate (GlcNAc-6-P) deacetylase and of *nagB* a glucosamine-6-phosphate (GlcN-6-P) deaminase (3,9,11–12). Recently, an additional gene locus has been reported for *B. subtilis* that encodes for the *gamAP* operon (13,14). Evidence has accumulated that this operon might be more specifically responsible for the uptake and catabolism of glucosamine, whereas the *nag* operon might be primarily responsible for GlcNAc utilization. The *gamAP* operon encodes for its own repressor molecule, named either YbgA or alternatively GamR, which shares about 31% sequence identity with NagR (13,14). While in the case of GamR it was possible to directly observe the detachment of the repressor from operator DNA upon addition of GlcN-6-P in electrophoretic mobility shift assays (EMSAs), similar experiments with NagR remained inconclusive (3,14). So far, NagR has been shown to bind GlcNAc-6-P with mM affinity, and the ligand-binding site could be mapped onto the structure of NagR by site directed mutagenesis (7). However, in the case of the homologous protein DasR from *S. coelicolor* (37% overall sequence identity to NagR) GlcN-6-P has been identified as the physiological effector (15). The residues surrounding the effector-binding site are almost completely conserved between NagR and DasR (see below). When considering that the genes of all three proteins GamR, NagR and DasR are embedded in operons encoding proteins responsible for the utilization of amino sugars, it seems likely that GlcN-6-P and/or GlcNAc-6-P are also effectors of NagR. At the same time, however, the possibility remains that slightly modified phosphorylated sugar compounds represent the physiological effectors of NagR.

NagR, GamR and DasR belong to the family of GntR transcription regulators and more specifically are members of the GntR/HutC subfamily (7,16). With more than 49000 deposited sequences, the GntR family of regulators (gluconate-operon repressor, Pfam family: PF00392) represents one of the largest groups of bacterial metabolite-responsive transcription factors (17,18). Their tertiary structure can be subdivided in almost all cases into two distinct domains: an N-terminal DNA-binding winged-helix-turn-helix (wHTH) domain and a C-terminal small-molecule effector-binding and oligomerization domain. Whereas the wHTH domain is structurally conserved throughout the GntR family, the architecture of the C-terminal domain diverges extensively among GntR family members and gives rise to four major subfamilies, named FadR, HutC, MocR and YtrA, which account for 43, 25, 16 and 11%, respectively, of all sequences currently available for GntR members (18). Thus, they encompass nearly 95% of all GntR members, whereas additional subfamilies like AraR are considerably less populated (8,16). In NagR and the entire GntR/HutC subfamily the C-terminal effector-binding domain adopts a so-called UTRA domain fold (Pfam family: PF07702) that is structurally homologous to the enzyme chorismate lyase (17). Concomitant with this observation, it has been proposed that the effector-binding site in the GntR/HutC members coincides with the position of the active site in chorismate lyase (19).

We previously described the first crystal structure of a full-length GntR/HutC member, namely of NagR from *B. subtilis* (7). In addition, a crystal structure of YydK also from *B. subtilis* (27% sequence identity with NagR) has been available from the protein database from a structural genomics initiative (PDB ID: 3BWG, (20)). These structures in combination with mutational analyses allowed us to propose a model for effector recognition and induction of NagR. In this model, a bound sulfate molecule mimics the phosphate group of the putative effector GlcNAc-6-P and is sandwiched between two helices in the effector-binding domain. Effector binding ultimately leads to a repositioning of the DNA-binding domains (DBD) with respect to the effector-binding domains with a ‘jumping-jack’-like motion (7). However, many issues concerning the allosteric coupling mechanism remained unresolved (7). Thus, so far, no structure of either a DNA-bound or effector-bound NagR or of any member of the extensive GntR/HutC subfamily has been reported. Nor is it understood how in detail effector binding modulates the DNA-binding affinity in these repressors.

Here, we present the first crystal structures of a full-length GntR/HutC member in complex with two putative effectors, namely of NagR in complex with GlcN-6-P and GlcNAc-6-P. We also report the first crystal structure of a GntR/HutC member in complex with DNA, namely of both full-length NagR and isolated NagR–DBDs in complex with a *dre*-site-derived double-stranded DNA (dsDNA) fragment. When combined with biophysical assays, these crystal structures provide a clear picture regarding the extraordinary large effector-induced conformational changes occurring in NagR and at the same time show to what extent proper NagR function depends on the fine-tuning of its DNA-binding affinity.

## MATERIALS AND METHODS

### Cloning, protein production and purification

A pET15b-vector (Novagen, EMD Biosciences, Darmstadt, Germany) containing the *nagR* gene was used as a template for the cloning of the isolated DBD of NagR (NagR–DBD, residues 1–75). The DNA fragment was amplified using the polymerase chain reaction with the forward primer DBD_fw (5′-GCGCGGCAGCCATATGAATATC-3′) and the reverse primer DBD_rev (5′-GCATAATGGATCCTCACTAGCTGACAAAGGTGCCCCGC-3′). Subsequent cloning into an empty pET15b-vector using restriction enzymes NdeI and BamHI (New England Biolabs, Ipswich, MA, USA) leads to the addition of an N-terminal hexahistidine tag and a thrombin cleavage site. Successful cloning was verified by DNA sequencing. Full-length NagR and truncated NagR–DBD were produced and purified as previously described for full-length NagR (21).

### Design and duplexation of dsDNA *dre*-site constructs

Various dsDNA constructs were designed for binding and crystallization studies (Supplementary Table S1). The 15mer native *nagAB* and *nagP* constructs contain the native *dre*-site sequences of NagR upstream of the *nagA-nagB-nagR* and the *nagP* operon in *B. subtilis*, respectively (9). The 15mer palindromic construct was derived from the consensus sequence of the two native non-palindromic *dre*-site sequences. All 15mer dsDNA constructs contain a 3′-T-overhang. The 19mer palindromic construct for crystallization consists of the 15mer palindromic sequence with two additional nucleotides at both the 5′- and 3′-end. The palindromic and native 26mer constructs for surface plasmon resonance (SPR) measurements contain the same motifs as the 15mer constructs, however with elongated boundary regions and an additional Biotin-tag at one 5′-end. Furthermore, the 26mer non-*dre*-site-containing control constructs comprise either the nucleotides 4 to 29 of the NagR-regulated gene *nagA* or are randomly designed. For DNA duplexation, the purified single-stranded 15mer and 19mer DNA constructs were diluted in 20 mM Tris–HCl pH 7.5, 150 mM NaCl, mixed at equimolar concentrations, heated to 95°C for 8 min and slowly cooled down to room temperature for successful annealing. The 26mer constructs were duplexed in 10 mM HEPES pH 7.4, 150 mM NaCl, 3 mM Ethylenediaminetetraacetic acid according to the same protocol.

### Analytical size exclusion chromatography

Analytical size exclusion chromatography (SEC) runs with NagR and NagR–DBD were performed in 20 mM Tris–HCl pH 7.5, 150 mM NaCl on a Superdex 200 10/300 GL and a Superdex 75 10/300 GL column (GE Healthcare, Munich, Germany), respectively. Concentrations of the dsDNA constructs were kept constant at 3 μM, while protein concentrations varied from 0 to 20 μM.

### Surface plasmon resonance measurements

SPR measurements with full-length NagR and NagR–DBD were performed on a BIAcoreX instrument at 25°C (BIAcore, Uppsala, Sweden) with subsequent data evaluation via the BIAevaluation software (GE Healthcare, BIAcore AB, Uppsala, Sweden). To analyze protein–DNA interactions, biotinylated 26mer dsDNA constructs were used for coupling onto a sensor chip SA (Supplementary Table S1) (GE Healthcare Bio-Sciences AB, Uppsala, Sweden).

For interaction studies with full-length NagR, 8 resonance units (RU) of the respective dsDNA construct (*dre*-site- or non-*dre*-site-containing) were coupled in flow cell 2, while flow cell 1 was left blank (not containing immobilized control dsDNA, as below) to serve as a reference surface. This procedure was valid, as NagR exclusively forms non-*dre*-site-specific complexes at concentrations above 25 nM (Supplementary Figure S1). Experiments with *dre*-site-containing dsDNA, however, were only performed up to NagR concentrations of 5 nM and therefore should not contain any non-*dre*-site-specific binding events. For studies with NagR–DBD and *dre*-site-containing DNA (palindromic and native), 76 and 88 RU of dsDNA, respectively, were immobilized in flow cell 2, while flow cell 1 served as a reference surface by coupling the same amounts of non-*dre*-site-containing control dsDNA (derived from *nagA*).

HBS-EP buffer (pH 7.4, GE Healthcare Bio-Sciences AB, Uppsala, Sweden) was used for sample dilution and as a running buffer during interaction analyses. Titrations for kinetic measurements with NagR and *dre*-site-containing DNA constructs were performed in triplicate in each case. The equilibrium constants of NagR toward non-*dre*-site-containing control DNA as well as of NagR–DBD toward *dre*-site-containing DNA were determined with SigmaPlot (Systat Software, San Jose, CA, USA) by Langmuir fits of plots from the steady-state response versus the analyte concentrations also from three different titrations. All quantitative measurements yielded reproducible data. For all measurements of NagR with *dre*-site-containing DNA, an additional regeneration step with 1 M NaCl was performed after each cycle to release NagR from the DNA on the chip surface.

### Crystallization of NagR complexes

Full-length NagR in complex with GlcN-6-P or GlcNAc-6-P was crystallized using the sitting-drop vapor-diffusion method by mixing 0.4 μl NagR (5.6 mg/ml NagR in 20 mM Tris–HCl pH 7.5, 150 mM NaCl, containing 10 mM GlcN-6-P or GlcNAc-6-P) with 0.2 μl reservoir solution and equilibrating the droplets against 50 μl reservoir solution at 292 K. Diffraction quality crystals were obtained with a reservoir solution containing 100 mM HEPES pH 7.5, 200 mM ammonium chloride and 25% (v/v) glycerol ethoxylate. The crystals were flash-cooled in liquid nitrogen using either 20% (v/v) ethylene glycol (NagR in complex with GlcN-6-P) or a mixture of 10% (v/v) glycerol and 10% (v/v) ethylene glycol (NagR in complex with GlcNAc-6-P) as cryoprotectant.

Prior to the crystallization of NagR in complex with 19mer palindromic dsDNA equal volumes of 100 μM NagR dimer and 100 μM dsDNA were mixed and incubated for 1 h at room temperature. The sample was then subjected to a preparative SEC run using a Superdex 200 10/300 GL column with 20 mM Tris–HCl pH 7.5, 150 mM NaCl as a running buffer. This step was performed to separate higher-oligomeric non-*dre*-site-specific protein–DNA complexes from the *dre*-site-specific 1:1 complex. The isolated complex peak was concentrated to a protein concentration of 5.5 mg/ml. Single crystals of the NagR–DNA complex were obtained by mixing 0.4 μl of the complex with 0.2 μl reservoir solution (0.1 M Bis-Tris pH 6.5, 25% (w/v) polyethylene glycol 3500) and equilibrating the droplets via the sitting-drop vapor-diffusion method against 50 μl reservoir solution at 292 K. Crystals were flash-cooled in liquid nitrogen using 30% (v/v) glycerol as cryoprotectant.

NagR–DBD in complex with 15mer palindromic dsDNA was crystallized using the sitting-drop method by mixing 0.2 μl NagR–DBD (5.9 mg/ml NagR–DBD in 20 mM Tris–HCl pH 7.5, 150 mM NaCl, containing 170 μM 15mer palindromic dsDNA) with 0.2 μl reservoir solution (50 mM sodium cacodylate pH 6.5, 200 mM sodium citrate, 10 mM MgCl_2_ and 5% (v/v) isopropanol) and equilibrating the droplets against 50 μl reservoir solution at 292 K. Crystals were flash-cooled in liquid nitrogen with 20% (v/v) 2-Methyl-2,4-pentanediol as cryoprotectant.

### Diffraction data collection, structure determination and refinement

Diffraction datasets of NagR in complex with GlcN-6-P or GlcNAc-6-P as well as of NagR and NagR–DBD in complex with palindromic dsDNA were collected from single crystals at 100 K at synchrotron beamline BL14.1 operated by the Helmholtz-Zentrum Berlin at the BESSY II electron storage ring (Berlin-Adlershof, Germany (22)) to resolutions of 2.0 and 2.05 Å for the complexes of NagR with putative effectors, and 2.9 Å as well as 1.9 Å for NagR and NagR–DBD in complex with dsDNA. Data were indexed and integrated with XDS and scaled with XSCALE (23,24). Initial phases for each dataset were determined via molecular replacement with PHASER (25) using the isolated DBD and effector-binding domain from a previous NagR structure (PDB ID: 2WV0, (7)) as search models. Molecular replacement solutions were readily obtained and the resulting models stepwise completed by multiple cycles of manual model building with COOT (26) and automated refinement with PHENIX (27). The molecules GlcN-6-P and GlcNAc-6-P were loaded via the LIBCHECK plug-in of COOT using their 3-letter codes GLP and 16G and were placed into unambiguous residual electron density. Analogously, ideal B-DNAs of the 19mer and the 15mer palindromic dsDNA sequences were built in COOT and modeled into clearly-defined difference density calculated with DNA-bound NagR and NagR–DBD diffraction data, respectively. The quality of the final models was validated with MolProbity (28). Crystallographic data collection and refinement statistics are summarized in Table 1. All structural illustrations were prepared with Chimera (29). Protein–DNA and protein–ligand interactions were analyzed using the analysis software NUCPLOT (30) and LigPlot+ (31), respectively. For comparison the structures were superimposed with program LSQKAB (32) of the CCP4 Software Suite (33). The sequence conservation in NagR-binding sites from *Bacillales* was illustrated with WebLogo 3.3 (34).

**Table 1.** Data collection and refinement statistics

Dataset	NagR + GlcN-6-P	NagR + GlcNAc-6-P	NagR + 19mer dsDNA	NagR–DBD + 15mer dsDNA
**Data collection^a^**				
Beamline	BESSY-MX, BL 14.1			
Wavelength (Å)	0.91841			
Resolution (Å)	35.00–2.05	35.00–2.00	35.00–2.90	35.00–1.91
	(2.12–2.05)	(2.07–2.00)	(3.01–2.90)	(1.98–1.91)
Space group	R32 :H	R32 :H	P4_3_2_1_2	P3_1_
Cell parameters (Å/°)	98.5/98.5/355.1	99.1/99.1/354.0	80.0/80.0/240.0	48.6/48.6/153.9
	90/90/120	90/90/120	90/90/90	90/90/120
Total reflections	182317 (17692)	512951 (50379)	127822 (12058)	120087 (11497)
Unique reflections	42017 (4067)	45784 (4525)	18046 (1754)	31230 (3069)
Redundancy	4.3 (4.4)	11.2 (11.1)	7.1 (6.9)	3.8 (3.7)
Completeness (%)	99.8 (98.6)	99.9 (99.8)	99.9 (99.8)	99.8 (98.7)
*I/σ(I)*	19.4 (2.4)	20.2 (2.4)	16.8 (1.2)	10.6 (1.9)
Wilson *B*-value (Å^2^)	34.8	36.0	90.1	25.8
*R*_merge_^b^ (%)	5.8 (65.6)	8.9 (126.4)	9.9 (197.9)	8.5 (86.5)
*R*_meas_^c^ (%)	6.6	9.4	10.7	9.9
CC_1/2_^d^ (%)	99.9 (74.6)	99.9 (90.5)	99.9 (29.4)	99.7 (67.8)
**Structure refinement**				
Resolution (Å)	32.65–2.05	32.73–2.00	34.28–2.90	32.52–1.91
*R*_work_/*R*_free_ (%)^e^	19.97/24.78	20.65/25.30	22.89/27.78	17.84/22.93
No. of non-hydrogen atoms	4284	4246	4350	3350
No. of protein residues	485	486	443	329
No. of solvent molecules	285	253	2	287
Additional molecules	2 × GlcN-6-P	2 × GlcNAc-6-P	1 × 19mer dsDNA	1 × 15mer dsDNA
	25 × ethylene glycol	13 × ethylene glycol	7 × ethylene glycol	2 × chloride ion
		2 × glycerol		
Average *B*-factors (Å^2^)	38.5 (all atoms)	41.3 (all atoms)	85.0 (all atoms)	29.9 (all atoms)
	38.0 (protein)	41.0 (protein)	85.3 (protein + DNA)	29.4 (protein + DNA)
	44.0 (water)	45.1 (water)	58.7 (water)	34.3 (water)
	42.1 (ligands)	44.4 (ligands)	81.1 (ligands)	22.9 (ligands)
Ramachandran favored (%)	98.5	98.6	96.0	98.3
Ramachandran outliers (%)	0.0	0.0	1.2	0.0
R.m.s. deviations				
Bonds lengths (Å)	0.008	0.008	0.011	0.007
Bond angles (°)	1.137	1.096	1.430	1.087

^a^Values for the highest resolution shell are listed in parentheses.

^b^*R*_merge_ = Σ|*I* − <*I*>|/Σ*I*, where *I* is the integrated intensity of a given reflection.

^c^*R*_meas_ is the multiplicity weighted merging *R*-factor.

^d^Correlation coefficient between two random halves of the dataset as described by Karplus and Diederichs (24), and calculated using XDS (23).

^e^*R*_work_ = Σ∥*F*_obs_| − |*F*_calc_∥/Σ|*F*_obs_|. *R*_free_ was calculated using 5% of data excluded from refinement.

## RESULTS

### Crystal structures of NagR in complex with GlcN-6-P and GlcNAc-6-P

**Figure 1.**
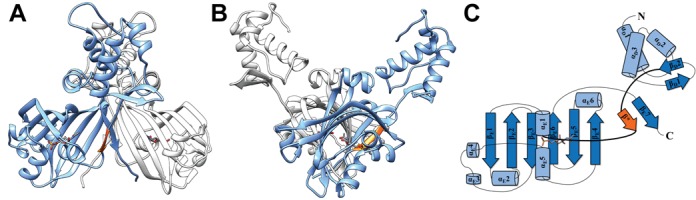
Crystal structure and topology plot of ligand-bound NagR. (**A**) Dimer of NagR in complex with GlcNAc-6-P in a cartoon representation with the monomers colored in blue and light gray and the ligand GlcNAc-6-P shown as a stick-model. (**B**) Side view of the complex after a 90° rotation. (**C**) Topology plot of monomeric NagR in complex with GlcNAc-6-P. Secondary structure elements are displayed as light blue cylinders (α-helices) and blue arrows (β-strands). The linker region between the DNA- and the effector-binding domain is highlighted in bold. The newly formed β-strand β* that appears upon ligand binding is colored in orange in all panels.

The crystal structures of the complexes between NagR and GlcN-6-P as well as NagR and GlcNAc-6-P were solved at 2.1 and 2 Å, respectively (Table 1, Figure 1A and B). The two complexes crystallized isomorphously and their asymmetric unit contains one entire NagR homodimer. Each monomer comprises a total of nine α-helices and ten β-strands and can be divided into a DBD and an effector-binding domain (Figure 1C). The peptide segment that interconnects the two domains is well defined by its electron density in each monomer (data not shown). Secondary structure elements are in the following referred to as either α_D_/β_D_ or α_E_/β_E_ in order to highlight the domain to which they belong. Strands β_E_1 to β_E_6 form the central β-sheet of the effector-binding domain and are involved in dimerization and ligand coordination. Superposition of the different structures shows that the average pairwise r.m.s. deviation between the Cα-atoms of all monomers in the two complexes present in the two asymmetric units is as small as 0.34 Å. This reflects not only the isomorphism of the crystals but even more importantly that the orientation of the DBDs with respect to the effector-binding domains is identical in all four molecules.

**Figure 2.**
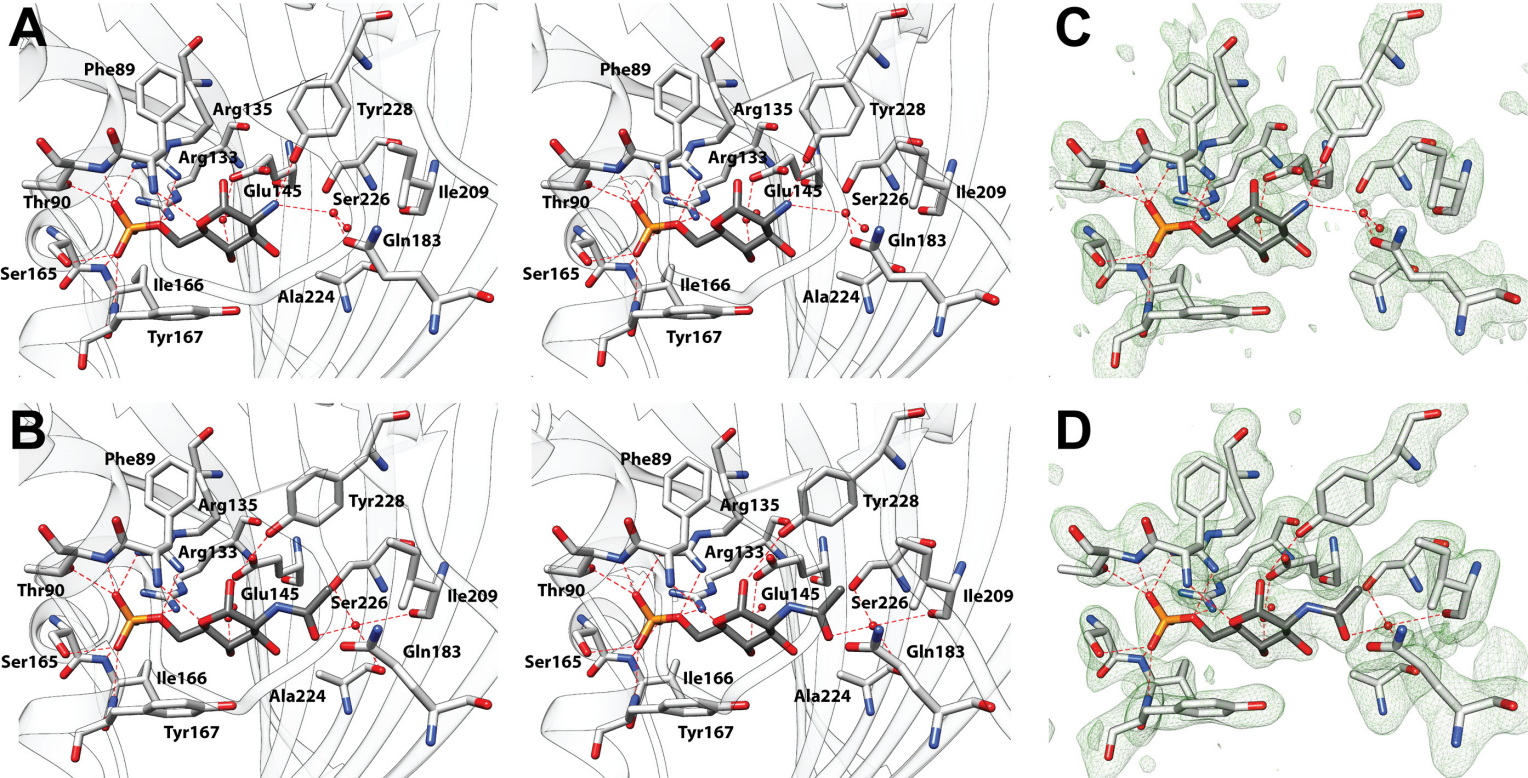
Closeup view of the effector-binding site of NagR. (**A**) and (**B**) Stereo view showing the interactions of (A) GlcN-6-P and (B) GlcNAc-6-P with NagR. Ligands and residues are presented as stick models and water molecules as red spheres. (**C**) and (**D**) Simulated annealing *F*_o_*-F*_c_ omit maps showing (C) the GlcN-6-P and (D) GlcNAc-6-P-binding site of NagR in the respective structures. Maps were calculated with PHENIX and are contoured as green mesh at 3.0 σ. Ligand molecules and all residues omitted during refinement and map calculation are shown. Chain B of the two NagR chains present in each crystal structure was selected arbitrarily for generating all the illustrations.

GlcN-6-P and GlcNAc-6-P are bound to NagR in an α-anomeric configuration. Each phosphate group is coordinated by the N-termini of helices α_E_1 (Thr90) and α_E_5 (Ser165, Ile166 and Tyr167) as well as by residues Arg133 and Arg135 presented by β-strand β_E_2 (Figures 1 and 2). Accordingly, NagR uses the half positive charges provided by the dipoles of helices α_E_1 and α_E_5 and the positive charge of both arginines for a stable coordination of the phosphorylated sugars. Such helix dipole interactions are very common for low molecular weight phosphate-containing ligands (35,36). The sugar moieties are predominantly recognized via direct or water-mediated hydrogen bonds involving polar main and side chain atoms from residues Phe89, Glu145 and Tyr228 as well as via hydrophobic interactions with Ser88, Arg211, Glu222 and Ala224. The phosphorylated sugars stack against Tyr167 and form an additional CH/π interaction often observed in sugar–protein complexes (37,38). Whereas the acetyl component of GlcNAc-6-P is indirectly contacted by Ile209 and Ser226, the resulting additional space in the binding pocket in the case of GlcN-6-P is filled by a water molecule and an adjacent ethylene glycol (EDO) molecule from the cryoprotectant solution. Altogether, the crystal structures reveal nearly identical interactions between the two phosphorylated sugars and NagR. However, the affinity of the two ligands for NagR is low, and in previous ITC experiments a *K*_D_ value of 1 mM was recorded for the binding of GlcNAc-6-P to NagR (7). Nevertheless, the two ligand are clearly defined by their electron densities and their binding interactions can be corroborated by the calculation of extended OMIT electron density maps (Figure 2C and D). At the same time, GlcN-6-P and GlcNAc-6-P binding appears to profoundly affect the domain organization of NagR since in the complexes the orientation of the DBD with respect to the effector-binding domain differs considerably from that observed in our previous sulfate-bound structure (7).

### Crystal structure of NagR in complex with operator DNA

**Figure 3.**
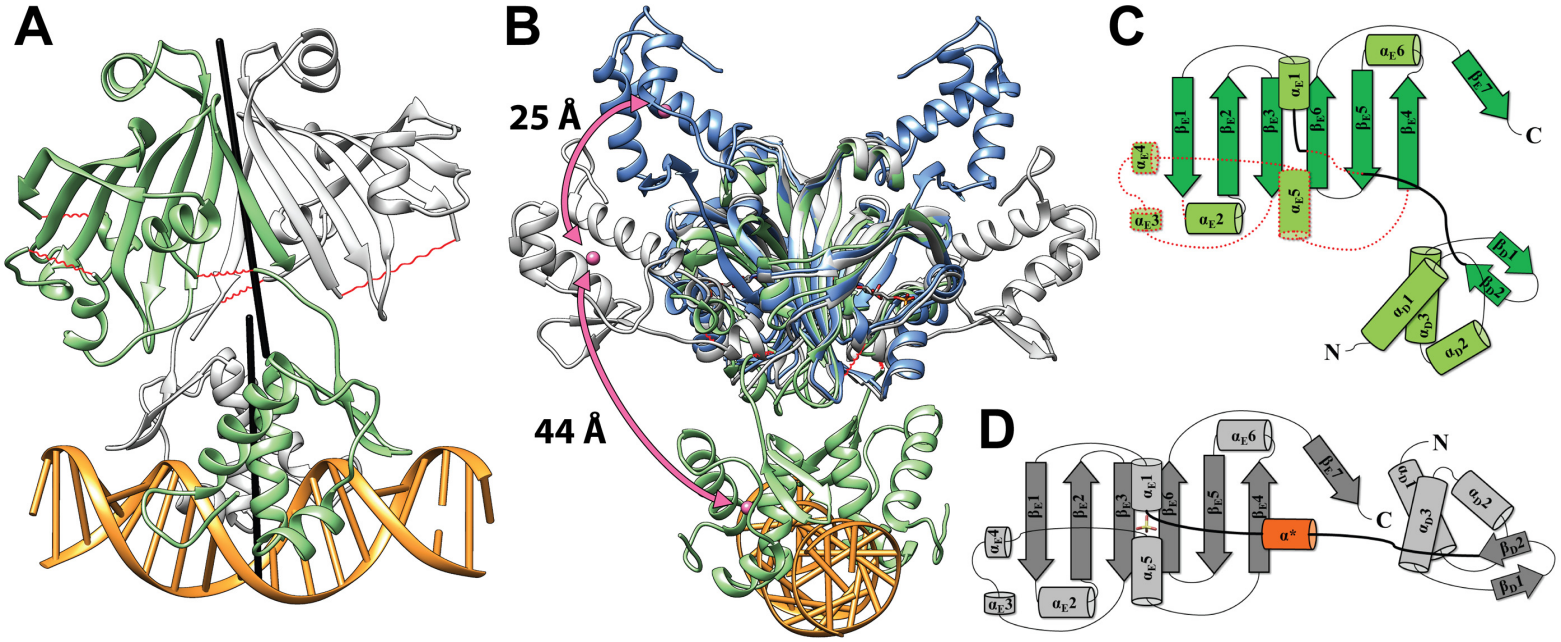
Crystal structure of wild-type NagR in complex with 19mer palindromic dsDNA. (**A**) Dimer of NagR in complex with palindromic dsDNA in a cartoon representation with the monomers colored in green and light gray. Segments with missing electron density in the structure of NagR are highlighted as red springs. The non-crystallographic symmetry axes relating the two protein chains in the DBD dimer as well as in the effector-binding domain dimer were generated with Chimera and are shown as black rods. (**B**) Superposition of the crystal structures of NagR in complex with palindromic dsDNA (green, PDB ID: 4WWC), sulfate-bound NagR (light gray, PDB ID: 2WV0) and GlcNAc-6-P-bound NagR (blue, PDB ID: 4U0W) in a cartoon representation. Sulfate molecules present in 2WV0 are omitted for clarity. The center of mass for one DBD of each dimer was calculated with Chimera and is presented as a pink sphere. (**C**) Topology plot of monomeric NagR in its DNA-bound conformation. Secondary structure elements and loop regions that are missing in chain A and/or B are marked with a red dotted line. (**D**) Topology plot of monomeric NagR in complex with a bound sulfate molecule (7). Helix α* that is only present in the sulfate-bound NagR structure is colored in orange. In panels (C) and (D) the secondary structure elements are displayed as cylinders (α helices) and arrows (β-strands). The linker region between the DNA- and the effector-binding domain is highlighted by a bold line.

To gain insight into DNA binding in the GntR/HutC subfamily of repressors, we determined the crystal structure of full-length NagR in complex with an idealized palindromic operator *dre*-site at 2.9 Å resolution. The asymmetric unit contains one NagR dimer in complex with a 19 bp fragment of dsDNA (Figure 3A). Electron density is missing for several segments in the effector-binding domain as well for the peptide segment that interconnects the effector-binding domain and the DBD in each chain A and B of homodimeric NagR. In detail, helices α_E_3, α_E_4 and α_E_5 are not formed in chain A, while chain B lacks most parts of helix α_E_3 as well as the N-terminal ends of helices α_E_2 and α_E_5. Regarding the interdomain linker, residues 79 to 83 and residues 81 to 85 are missing in chain A and B, respectively. Apart from the striking absence of beta-strand β*, which occurs in the interdomain linker region of the GlcN-6-P and GlcNAc-6-P-bound state of NagR (Figure 1), DNA-bound NagR features a highly similar topology consisting of nine α-helices and nine β-strands (Figure 3C).

In the DNA-bound complex, the DBDs of NagR are pointing downward in direction of the very C-terminal strand β_E_7 (Figure 3A), which is in agreement with our previously proposed NagR DNA-binding model derived from YydK (7). Surprisingly, the orientation of the two-fold symmetry axis that relates the two effector-binding domains in NagR does not coincide with the two-fold axis that relates the two DBDs bound to the 19 bp palindromic DNA fragment since a crossing angle of 6.5° can be observed between the two local symmetry axes (Figure 3A). Thus it appears that in the DNA-bound and ligand-free structure of NagR not only secondary structure elements in the effector-binding domain are considerably less well defined but that also the DBDs are more flexibly attached to the effector-binding domain than in GlcN-6-P- and GlcNAc-6-P-bound NagR. Conversely, GlcN-6-P and GlcNAc-6-P binding appears to enhance the folding of the effector-binding domain and to lock the DBD more firmly to the latter domain.

### High resolution crystal structure of the complex between NagR–DBD and operator DNA

In order to obtain more detailed insight into the DNA-binding determinants of NagR, we also solved the crystal structure of isolated NagR–DBDs in complex with an idealized 15mer palindromic operator *dre*-site at a resolution of 1.9 Å. In contrast to the classical helix-turn-helix motif that is responsible for DNA binding in the TetR family of repressors, the wHTH domains in GntR repressors form stable domains, when produced in a discrete form (7). Thus, a fragment encompassing residues 1 to 75 of NagR gives rise to a characteristic protein CD spectrum and unfolds only slightly less cooperatively than full-length NagR in a thermal unfolding experiment (Supplementary Figure S2). The DBDs of AraR, a member of the eponymous GntR/AraR subfamily of GntRs, also form stable entities by themselves, and this property of wHTH domains might extend to other proteins (39,40).

**Figure 4.**
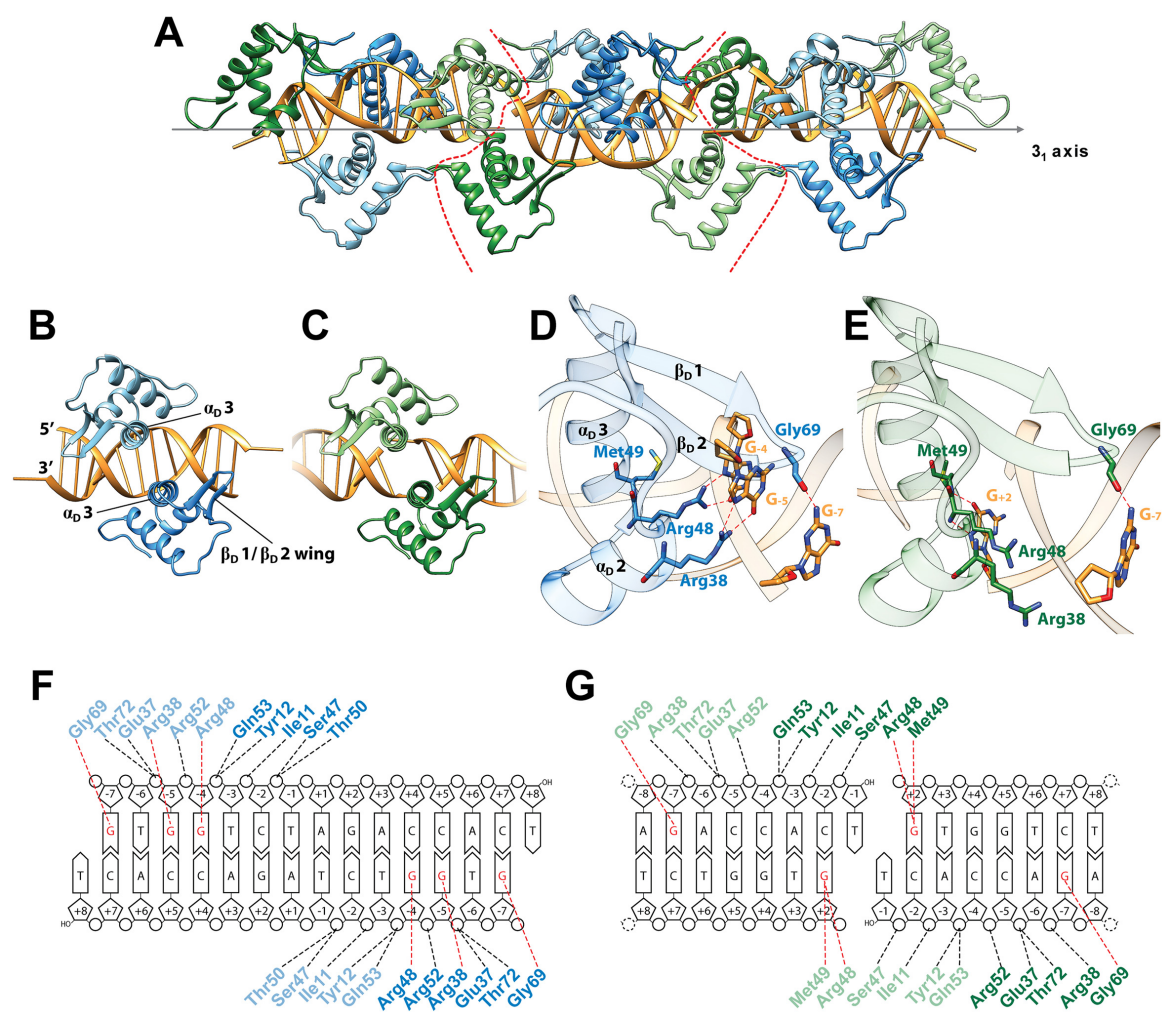
Crystal structure of NagR–DBD in complex with 15mer palindromic dsDNA. (**A**) The crystallographic asymmetric unit (ASU) comprises four NagR–DBD monomers bound to dsDNA (middle; delineated by red dotted lines). The complex is shown together with two adjacent ASUs that are related by a crystallographic 3_1_ screw axis as indicated. All molecules are shown in a cartoon representation with the centrally-bound NagR–DBD dimer colored in blue and light blue. The edge-bound NagR–DBD dimer is depicted in green and light green. (**B**) and (**C**) Close-up view of (B) a centrally-bound NagR–DBD dimer bound to dsDNA forming the *dre*-site-specific recognition complex and of (C) an edge-bridging NagR–DBD dimer that reveals non-*dre*-site-specific binding interactions. (**D**) and (**E**) Details of the interactions between DNA and (D) a centrally-bound NagR–DBD and (E) an edge-bound NagR–DBD. Only base-directed interactions are shown. Hydrogen bonds are represented by red dotted lines. Interacting residues and bases are shown as stick models. (**F**) and (**G**) Schematic summary of the NagR–DBD–DNA contacts formed by (F) the centrally-bound dimer and (G) the edge-bound dimer. Only direct interactions, identified with the analysis software NUCPLOT (30), are shown. Base-specific contacts are indicated in red. Nucleotides in the recognition half-sites are numbered according to their position from the center of the palindrome.

Although the dsDNA fragment used for crystallization was designed to contain only a single palindromic *dre*-site, we observe not two but four NagR–DBD molecules bound to the dsDNA fragment in the structure of the complex (Figure 4A). While two NagR–DBD molecules bind at the center of the dsDNA (as seen in the crystal structure of full-length NagR in complex with dsDNA (Figure 3A)), two additional NagR–DBDs bind at the edges of the dsDNA fragment. Interestingly, the two edge molecules pair up with symmetry-related copies from adjacent dsDNA fragments, and their overall DNA-binding mode is identical to that of the NagR–DBD molecules bound at the center of the dsDNA fragment (Figure 4B and C). The intermolecular interactions between the four NagR–DBDs reflect the crystal packing of the molecules, since in the crystal the dsDNA segments form a pseudo contiguous double helix that coincides with the crystallographic 3_1_ screw axis (Figure 4A).

Despite the fact that a superposition of the Cα-positions of all NagR–DBD monomers in the asymmetric unit yields an average pairwise r.m.s. deviation as low as 0.41 Å, the individual contacts formed with the DNA differ considerably between both complexes. As described below, we propose that the two centrally bound NagR–DBDs show how NagR highly specifically recognizes DNA operator sites, while the two NagR–DBD molecules located at the edges of the dsDNA fragment reflect a considerably less specific interaction mode.

The NagR–DBDs display the typical wHTH domain fold, which consists of three alpha helices α_D_1 (residues 11–24), α_D_2 (residues 37–44) and α_D_3 (residues 48–60) followed by strand β_D_1 (residues 64–67), the wing motif formed by the loop that connects beta-strands β_D_1 to β_D_2, and strand β_D_2 (residues 71–74) (Figure 1C). Although wHTH domains have been described that contain an additional third β-strand and a second wing motif, these features are absent in NagR and in many recent structures of wHTH domains (39–41). Thus, in NagR the previously postulated wHTH-typical third β-strand lacks clear β-conformation and pairs via only a single hydrogen-bond with strand β_D_2. In the DNA-bound complex the DBDs bind symmetrically side-by-side within the same contiguous major groove segment when viewing the DNA in a two-dimensional projection (Figure 4B). The two so-called recognition helices α_D_3 are juxtaposed to each other and oriented almost perpendicular to the dsDNA axis. They contact the DNA only via residues from their N-terminal side. At the same time, the wing region of each DBD reaches across the DNA-backbone and interacts with residues from the adjacent minor groove.

The centrally bound NagR–DBDs interact closely with the sequence-specific recognition half-sites of the palindromic dsDNA with the sequence 5′-GTGGTCTAGACCAC-3′. The recognition half-sites (underlined in the preceding sequence) are separated by six nucleotides and extend from positions −7 to −4 and +4 to +7, when numbered from the center of the palindrome. While each NagR–DBD molecule contacts two contiguous segments of dsDNA, namely the nucleotides −7 to −4 from one strand and −3 to −1 from the opposite strand, sequence-specific interactions are restricted to the −7 to −4 segment and more precisely to bases G_−7_, G_−5_ and G_−4_ (Figure 4D and F). All interactions with the nucleotides −3 to −1 from the opposite strand solely involve atoms of the sugar-phosphate backbone. Interestingly, the short 4-nucleotide-long segment −7 to −4 interacts with NagR via both its major and minor groove (Figure 4D). Thus, the wing motif reaches into the minor groove and contacts G_−7_ via the carbonyl oxygen of Gly69. We consider this contact to be protein sequence-specific as well since the wing motif forms a type-II β-turn with a strong preference for glycines at β-turn position i + 2 (42). Replacement of Gly69 would very likely perturb the conformation of the wing and abrogate this interaction. The bases G_−5_ and G_−4_ are specifically recognized by Arg38 and Arg48, respectively. While Arg38 is located within the first helical turn of helix α_D_2, Arg48 extends from a similar position in helix α_D_3. Both arginine residues form bidental contacts with the guanine base they recognize, and the terminal side chain nitrogen atoms contact guanine atoms O6 or N7. These molecular interactions are in full agreement with those observed for full-length NagR bound to DNA as observed in the 2.9 Å resolution crystal structure (Supplementary Figure S3). Taken together, recognition of G_−5_ by Arg38, G_−4_ by Arg48 and the interaction of the wing motif with the minor groove appear to represent the hallmarks of the specific interaction of NagR with DNA.

The DBDs bound at the edges of the dsDNA fragment lack most base-specific interactions since only the wing anchoring via Gly69 is conserved (Figure 4E). When applying an identical base numbering as used for bases contacted by the centrally bound DBDs, it becomes apparent that the bases at positions −5 and −4 are both substituted against cytosine (Figure 4G). Concomitantly, Arg38 and Arg48 of these DBDs no longer directly contact these bases and point either away from the interface or contact water molecules (and a chloride ion) that are trapped in the protein–DNA interface in the major groove. No novel side chain specific interactions are observed in this complex, however two previously unobserved main chain contacts, namely between the main chain nitrogens of Arg48 and Met49 with a guanine located at position +2 are formed (Figure 4E).

Binding of NagR–DBDs toward the edges of the dsDNA fragment does not appear to represent a mere crystallization artefact since a similar bridging effect can also be observed in solution. To show this, we designed a dsDNA fragment in which the high affinity-binding half sites with the GTGG motif were shifted toward the two 3′ edges of the fragment by interchanging the nucleotides at positions −7 to −2 with those at positions +2 to +7 (Supplementary Table S1, Figure 4F). Incubation of NagR–DBDs with these fragments immediately leads to the formation of higher molecular weight oligomers as monitored in EMSAs, thus demonstrating that the NagR–DBDs are able to bridge adjacent dsDNA fragments in solution provided that they bind to these with high affinity (Supplementary Figure S4).

We propose that the binding mode observed for the NagR–DBDs bound toward the edges of the dsDNA fragment in the crystal structure reflects how NagR is able to interact with less specific DNA sequences (see below). In this context it is interesting to note that all naturally occurring *dre*-sites in *B. subtilis* are not perfectly palindromic (Supplementary Table S2). In the *B. subtilis dre*-sites only one half-site contains two consecutive guanines at positions −5 and −4, whereas the second half-site contains only a single guanine. Thus, a mixed interaction model that combines the binding modes observed for the centrally-bound and the edge-bound NagR–DBDs might describe best the specific binding of NagR to naturally occurring non-palindromic *dre*-sites in *B. subtilis.*

### Amino sugar phosphate versus DNA binding to NagR reveals extraordinary large domain reorientations

When comparing the crystal structures of ligand-bound NagR with the structure of NagR in complex with an idealized palindromic operator *dre*-site and with the previously determined structure of sulfate-bound NagR, it becomes apparent that in all these structures the DBD adopt a wide range of different orientations (Figure 3B) (7). In the DNA-bound state, as shown above, the two DBD moieties of dimeric NagR are juxtaposed and point ‘downward’, namely into the same direction as the very C-terminal β-strand β_E_7 of the regulator. Upon binding of sulfate, the two DBDs of NagR are separated and move about 44 Å upward into an equatorial position (Figure 3B and D). However, upon binding of GlcN-6-P and GlcNAc-6-P, the DBDs are displaced even further by an additional 25 Å. They now point ‘upwards’ and are positioned close to helix α_E_6 (Figures 1C and 3B).

At the same time the repositioning of the DBDs goes hand in hand with distinct changes in the secondary structure elements in the effector-binding domain. In DNA-bound NagR, the DBDs appear to be only loosely associated with the effector-binding domain and several loops and secondary structure elements are partially disordered. In the sugar-bound structures all linker regions in the effector-binding domain are well defined and, most strikingly, helices α_E_1 and α_E_5 move closer to each other and coordinate the phosphate group of the bound ligands. We previously postulated a loop-to-helix transition and the formation of helix α* that forces the DBD to move apart upon ligand binding (Figure 3D) (7). The ligand-bound structures, however, now reveal an even more distinctive rearrangement of the DBDs and the formation of a novel beta-strand β* that interacts with the C-terminal strand β_E_7. This newly formed beta-strand β* appears to lock the DBD in the observed ‘upward’ positioning (Figure 1C). Overall, binding of phosphorylated sugars appears to engender distinct secondary structure rearrangements in the effector-binding domain and concomitantly large displacements of the DBDs with respect to the effector-binding domains of NagR.

### Both full-length NagR and isolated DNA-binding domains bind DNA with high affinity

**Figure 5.**
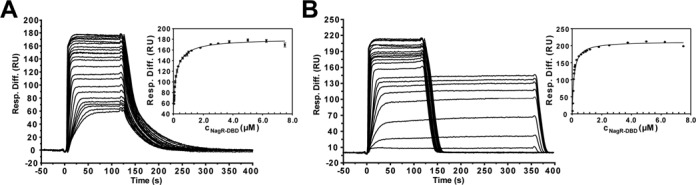
Quantitative analysis of NagR–DBD interactions with *dre*-site-containing dsDNA constructs. Sensorgrams from SPR analyses of the interaction of NagR–DBD with (**A**) palindromic and (**B**) native *nagAB* dsDNA for the respective triplicate measurements are shown. The corresponding diagrams for the determination of *K*_D_ values are also included and display the SPR response units plotted versus the NagR–DBD concentrations which were fitted to the Langmuir equation for a 2:1 and a 1:1 binding reaction, respectively. The error bars indicate the standard deviation among triplicate data. The concentrations of NagR–DBD ranged from 20 nM to 7.5 μM for palindromic dsDNA and from 25 nM to 7.5 μM for native *nagAB* dsDNA.

**Figure 6.**
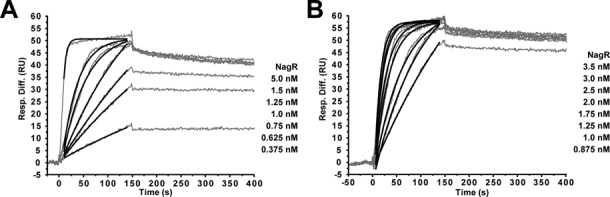
Quantitative analysis of NagR interactions with *dre*-site-containing dsDNA constructs. Sensorgrams from SPR analyses of the interaction of NagR with (**A**) palindromic and (**B**) native *nagAB* dsDNA of the respective triplicate measurements are shown. Kinetic analysis showed best fit to a 1:1 Langmuir type of interaction. From this interaction model the association rate constants (*k*_a_, corresponding fits shown as black curves) and dissociation rate constants (*k*_d_) were determined with values of *k*_a_ = 1.4 ± 0.3 × 10^7^ M^−1^s^−1^, *k*_d_ = 2.3 ± 0.4 × 10^−4^ s^−1^ and *k*_a_ = 1.8 ± 0.7 × 10^7^ M^−1^s^−1^, *k*_d_ = 1.7 ± 0.4 × 10^−4^ s^−1^ for the interaction with palindromic and native *nagAB* dsDNA, respectively. These values were used to calculate the equilibrium dissociation constants (*K*_D_) reported in Table 2. The NagR concentrations used are listed on the right side of the respective sensorgram.

In order to quantify the DNA-binding behavior of full-length NagR as well as of isolated NagR–DBDs we performed EMSAs and SPR measurements. In EMSAs both full-length NagR and NagR–DBD readily bind idealized palindromic *dre*-sites as well as naturally occurring *dre*-sites from *B. subtilis* and form discrete complexes (Supplementary Figure S5). Clear differences between NagR–DBDs and of full-length NagR become apparent in SPR experiments (Figures 5 and 6). Whereas individual NagR–DBDs bind idealized palindromic and non-palindromic *dre*-sites with nanomolar affinity, full-length dimeric NagR binds these DNA fragments with picomolar affinity in agreement with the expected avidity effect (Table 2).

**Table 2.** Equilibrium dissociation constants of NagR–DBD- and NagR-interactions with various DNA constructs obtained via SPR measurements

Protein	DNA construct	*K*_D_ (M)
NagR–DBD	26mer palindromic dsDNA	14.5 ± 3.3 × 10^−9^ (*K*_D1_)
		358.8 ± 43.9 × 10^−9^ (*K*_D2_)
NagR–DBD	26mer native *nagAB* dsDNA	122.3 ± 7.6 × 10^−9^
NagR	26mer palindromic dsDNA	27.9 ± 6.7 × 10^−12^
NagR	26mer native *nagAB* dsDNA	19.2 ± 15.1 × 10^−12^
NagR	26mer control dsDNA (derived from *nagA* gene)	127.0 ± 4.2 × 10^−9^
NagR	26mer control dsDNA (random design)	306.1 ± 6.3 × 10^−9^

A more detailed analysis of the binding affinity of NagR–DBD to idealized palindromic versus native non-palindromic *dre*-sites shows that in the former case the Langmuir binding isotherm can be best explained using a two-step binding model with *K*_D1_ = 14.5 ± 3.3 nM and *K*_D2_ = 358.8 ± 43.9 nM (Figure 5A), whereas in the latter case a single binding step model appears adequate (*K*_D_ = 122.3 ± 7.6 nM) (Figure 5B).

In the case of full-length NagR, we performed a kinetic analysis of the SPR sensorgrams and derived *K*_D_ = 27.9 ± 6.7 pM for the binding of NagR to the palindromic dsDNA construct (Figure 6A) and *K*_D_ = 19.2 ± 15.1 pM for the binding of NagR to the non-palindromic native *nagAB* dsDNA (Figure 6B). The SPR-deduced binding affinities of NagR are considerably higher than the previously observed nanomolar binding in ITC experiments (7). However, increased DNA-binding affinities of NagR have also been recently suggested by Gaugué *et al.* based on EMSA and DNaseI-footprinting experiments (14).

**Figure 7.**
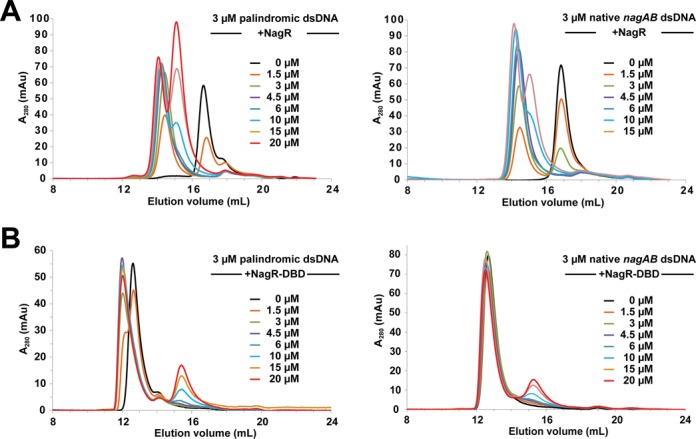
Analytical size exclusion chromatography runs of (**A**) NagR and (**B**) NagR–DBD showing the interaction with various 15mer dsDNA constructs derived from *Bacillus subtilis dre*-sites. The final concentrations of dimeric NagR and monomeric NagR–DBD are indicated for each curve. The dsDNA constructs were used with a final concentration of 3 μM.

We also investigated DNA binding using SEC and again discrete complexes are formed for full-length NagR in complex with palindromic and non-palindromic *dre*-sites (Figure 7A). However, here, we observe a clear difference in the binding behavior of NagR–DBD. In gel filtration experiments we detect the formation of a higher molecular weight complex exclusively when we incubate NagR–DBDs with idealized palindromic dsDNA and not in the case if NagR–DBDs are mixed with non-palindromic *dre*-site-encoding dsDNA (Figure 7B). Since both DNA fragments display nanomolar-binding affinities in SPR measurements, this observation seems puzzling. However, it has to be considered that a gel filtration chromatography experiment does not proceed under equilibrium conditions since complexes of different stoichiometry migrate with different velocities thus continuously altering local concentrations. Therefore, this observation might hint to differences in the dissociation kinetics of NagR–DBDs bound to either palindromic or non-palindromic *dre*-sites. These differences should also apply to full-length NagR and might provide hints for why in *B. subtilis* only non-palindromic *dre*-sites are observed.

**Figure 8.**
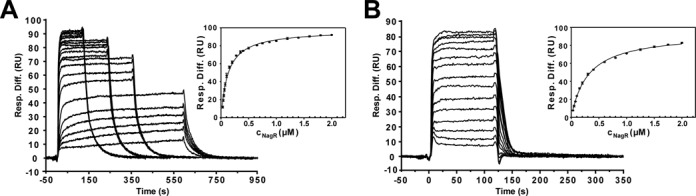
Quantitative analysis of the interaction of NagR with non-*dre*-site control dsDNA fragments. Sensorgrams from SPR analyses of the interaction of NagR with (**A**) a *nagA* gene derived and (**B**) a randomly designed control dsDNA of the respective triplicate measurements are shown. The corresponding diagrams for the determination of *K*_D_ values are also included and display the SPR response units plotted *versus* the NagR concentrations. A Langmuir 1:1 binding model was applied. The error bars indicate standard deviations between triplicate data. The corresponding NagR concentrations ranged from 30 nM to 2 μM for both dsDNA constructs.

SPR experiments also provide evidence that full-length NagR displays considerable affinity for random DNA sequences. Full-length NagR binds to non-*dre*-site-specific control DNA fragments derived either from a randomly chosen segment of the *nagA* gene (*K*_D_ = 127.0 ± 4.2 nM) (Figure 8A) or an otherwise randomized DNA fragment (*K*_D_ = 306.1 ± 6.3 nM) (Figure 8B) with nanomolar affinity. This is in agreement with the formation of higher molecular weight complexes that can be observed in EMSA experiments when the NagR/DNA ratio is gradually increased (Supplementary Figure S5A). This behavior has also been recently observed in DNaseI-footprinting experiments (14). In stark contrast, however, no binding is observed when testing control DNA fragments for the binding of isolated NagR–DBDs (Supplementary Figure S6). Thus, full-length NagR but not isolated NagR–DBDs, displays considerable affinity for random DNA sequences. At the same time, however, these affinities are about 1000-fold lower than the affinity of full-length NagR for *dre*-site-specific sequences.

## DISCUSSION

### The DNA-binding mode of NagR highlights shared features in GntR repressors

In general, wHTH domains bind dsDNA in different orientations and display highly diverse modes of interaction with DNA (40). However, this seems not to be the case for the wHTH domain in GntR repressors. The orientation of all four NagR–DBD molecules is identical in the high resolution crystal structure of the DNA complex. Moreover, this mode of interaction is highly similar to that observed in GntR members belonging to different subfamilies, namely in FadR and AraR (39,43–44). In all these structures two DBDs bind symmetrically side-by-side within a contiguous major groove segment. The so-called recognition helices α_D_3 from two DBDs are juxtaposed and bind perpendicular to the dsDNA axis. Helix α_D_3 as well as adjacent helix α_D_2 are important for the base-specific readout. At the same time the wing motif reaches across the DNA-backbone and interacts with the nucleotide bases via the minor groove.

The crystal structure of the GntR family member FadR from *Escherichia coli* in complex with its operator sequence solved at 3.2 Å resolution reveals a base-specific interaction of two arginines (Arg35 and Arg45) with two consecutive guanine bases similar to that observed for NagR (43,44). In contrast, in the recently published structures of the DBDs of AraR from *B. subtilis* in complex with two native non-palindromic operator sequences reported at resolutions between 2.0 and 2.3 Å only one of the two arginines is conserved, namely Arg41, which again specifically interacts with a guanine base (39). As is the case for Arg45 in FadR and Arg48 in NagR, this arginine is presented from the N-terminal side of helix α_D_3 and interacts with a guanine base that is equivalent to G_−4_ in the NagR operator complex.

Differences among these GntR members, however, exist regarding the number of nucleotides that separate the two sequence-specific recognition half sites. Whereas in *B. subtilis dre*-sites the recognition half-sites are separated by six nucleotides, the spacing only comprises five nucleotides in FadR and either six or eight nucleotides in the two different DNA–AraR complexes (39,43–44). In the case of AraR, these variations have been suggested to constitute an *in vivo* strategy for the modulation of the gene expression levels (39).

The combined findings show that despite profound differences, the GntR family members display common features regarding the molecular determinants for the base-specific recognition of DNA operator sequences. The structural information described here and in previous reports should therefore facilitate the modeling and understanding of the DNA operator specificity of a large number of GntR repressors.

### Non-palindromic *dre*-sites as a prerequisite for proper NagR function

**Figure 9.**
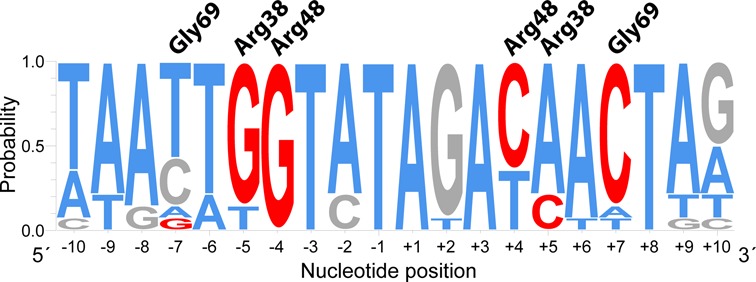
Sequence conservation in NagR transcription factor-binding sites in *Bacillales.* The nucleotide preference was calculated from the binding site sequences listed in Supplementary Table S2 and was generated with WebLogo 3.3 (34). GC bases are colored in gray, AT bases in blue. Nucleotides anticipated to participate in sequence-specific transcription factor binding are marked in red. Gly69 (wing motif), Arg38 (α_D_2) and Arg48 (α_D_3) are the NagR residues involved in base-specific DNA binding. These residues bind either to the listed segment or to the complementary strand.

It is remarkable that, unlike the dsDNA fragments that were used for the crystallization of NagR and NagR–DBD in complex with DNA, all naturally occurring *dre*-sites in *Bacillus* species are not perfectly palindromic (Supplementary Table S2). Strikingly, of the two consecutive guanine bases at positions −5 and −4 that are recognized by base-specific interactions with Arg38 and Arg48 in the perfectly palindromic dsDNA, only one guanine base occurs concurrently in both half-sites in native *dre*-sites. While G_−4_ is mostly conserved, G_−5_ is often replaced by thymine in one of the two half sites. Furthermore, the guanine base at position −7 that appears to anchor the wing motif via Gly69 is only conserved once per native *dre*-site, namely almost exclusively in the half site that lacks two consecutive guanine bases (Figure 9).

According to *in silico* studies by Marabotti *et al.*, a guanine-to-adenine substitution together with a loss of interaction with an arginine side chain should considerably reduce the affinity of a protein for DNA (45). However, we observe highly similar affinities for the binding of NagR to either palindromic or non-palindromic *dre*-sites in SPR experiments. Moreover and somehow counterintuitively, we observe that while binding of NagR–DBD molecules to palindromic sequences is described best by a two-step-binding model, binding of NagR–DBD to non-palindromic sequences can be satisfactorily modeled as a single binding event. Thus, it seems likely that indirect effects compensate for the loss of the Arg38 interaction in the second half site. In both crystal structures, NagR and NagR–DBD binding to the palindromic dsDNA fragment appear to go hand in hand with a distortion of the canonical B-DNA conformation (data not shown). This conformational adaptation might be more easily accomplished or, likewise, less pronounced for the binding to non-palindromic DNA sequences and this could explain both the one-step binding mechanism and why the absence of the second arginine–guanine interaction in one half-site does not reduce the overall binding affinity.

Whereas under equilibrium conditions, we don't observe any pronounced differences in the affinities of NagR–DBD for palindromic versus non-palindromic DNA sequences, it is striking that in gel filtration experiments complexes between NagR–DBD and DNA can only be observed in the case of palindromic and not in the case of naturally occurring non-palindromic *dre*-sequences. When taking into consideration the non-equilibrium nature of a gel filtration experiment, this behavior of NagR–DBD might hint to differences in the dissociation kinetics of the different complexes. Although we fail to corroborate this behavior with full-length NagR in SPR experiments, differences in the binding kinetics might be required for the proper functioning of NagR in a cellular context.

### Extraordinary large domain rearrangements fine-tune the DNA-binding properties in NagR and these rearrangements possibly extend to other GntR/HutC family members

We propose that the crystal structures presented here map the two functionally most important conformational states of NagR, namely the operator DNA-bound and the effector-bound state of NagR. The structure of DNA-bound NagR represents the first crystal structure of a GntR/HutC family member bound to DNA and is in full agreement with the previously postulated YydK-derived model of the DNA-competent NagR conformation (7). The structure also corroborates our previous biochemical studies on the DNA-binding properties of a conformationally locked NagR mutant (NagR-E61C-L242C) (7). Moreover, the additional DNA-bound complex of the NagR–DBDs provides detailed insight into base-specific versus non-specific DNA recognition.

At the same time the structures of GlcNAc-6-P- and GlcN-6-P-bound NagR reveal that ligand binding goes in hand with distinct secondary structure rearrangements in the effector-binding domain and an extraordinary large repositioning of the DBDs with respect to the effector-binding domains. The presented crystal structures are also in full agreement with a previous mapping of the NagR ligand-binding site via site-directed mutagenesis (7). As mentioned in the introduction, there is room for discussion whether these sugars qualify as biological effectors of NagR since they only bind with millimolar affinity. In addition, we and others failed to observe a direct detachment of NagR from DNA upon addition of either GlcNAc-6-P or GlcN-6-P (data not shown, (14)) However, for the reasons described below, we propose that the structures presented here either comprehensibly mimic or directly represent the effector-bound induced state of NagR. Thus, in the case of the homologous protein DasR from *S.* *coelicolor* (37% overall sequence identity to NagR) GlcN-6-P has been identified as the physiological effector (15). A sequence alignment between NagR and DasR shows that 8 out of 12 residues that contact either GlcNAc-6-P or GlcN-6-P in NagR are fully conserved in DasR with the remaining four residues being highly similar (Supplementary Figure S7). This suggests that the ligand-binding mode observed in NagR extends to DasR. At the same time, DasR and NagR participate in the regulation of highly similar *dre*-sites upstream of genes for GlcNAc uptake and metabolism in either *B. subtilis* or *S. coelicolor* (3). When taken together, it seems likely that these molecules share similar effector molecules.

Our previous investigations lead to the postulation of a ‘jumping jack’ model for the induction mechanism of NagR. According to this model NagR toggles between two conformations, namely a DNA-binding competent conformation in which the two DBD heads in NagR are juxtaposed and a significantly less competent conformation that is induced after binding of the small molecule ligands GlcNAc-6-P and GlcN-6-P to NagR. The here presented novel structures suggest that the previous sulfate-bound NagR conformation might represent a mere intermediate between the DNA-bound and the effector-bound conformation of NagR. Concomitantly, the previously postulated loop-to-helix transition in the effector-binding site might actually correspond to a loop-to-helix-to-strand transition (Figures 1 and 3).

Interestingly, the positioning of the DBDs in the GlcNAc-6-P- or GlcN-6-P-bound NagR structures does not *per se* exclude DNA binding. Indeed, it is possible to juxtapose two effector-bound NagR dimers and superimpose them onto the structure of isolated NagR–DBDs in complex with dsDNA so that each NagR dimer contacts the DNA via a single DBD (Supplementary Figure S8). At the same time our binding studies with isolated NagR–DBDs suggest that simultaneous binding of two NagR dimers would result in nanomolar binding affinity for *dre*-site-specific DNA whereas no binding of DBD pairs belonging to different NagR dimers should be observed for the binding to random DNA sequences. The formation of such a *dre*-site-specific pentameric complex would readily explain available *in vitro* and *in vivo* data. Thus it has been observed that full derepression is difficult to achieve in *B. subtilis*, as *nagR*-deletion mutants grow better on GlcNAc than the wild type strain (13). Furthermore, Gaugué *et al.* recently showed via combined EMSA and DNaseI-footprinting experiments that neither GlcNAc-6-P nor GlcN-6-P at high concentrations (up to 10 mM) induces a detachment of NagR from its native *dre*-site sequences (14). These observations suggest that even in the induced state NagR still retains considerable operator-binding properties.

**Figure 10.**
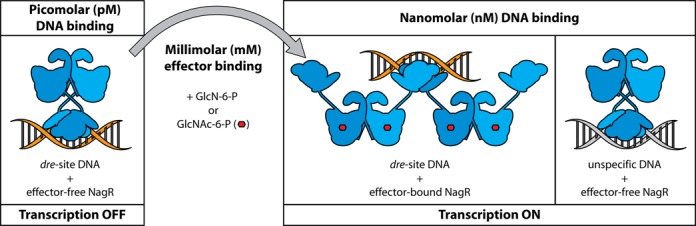
Effector binding and concomitant structural rearrangements modulate DNA binding in NagR. Graphical summary of the DNA-binding modes and affinities observed for NagR. Dimer-forming monomers of NagR are illustrated schematically in blue. Effector molecules are depicted as red hexagons. *Dre*-site-specific and unspecific dsDNA is colored in orange and gray, respectively.

We anticipate that the formation of such a pentameric complex consisting of two effector-bound NagR dimers and a *dre*-site-specific DNA fragment would, however, not preclude gene transcription since its DNA-binding affinity would be similar to the affinity of effector-free full-length NagR for random DNA-sequences (Figure 10). As shown above, full-length NagR binds random DNA sequences also with nanomolar affinity in comparison to the picomolar affinity for *dre*-site-specific DNA binding. Moreover, these analyses suggest that lowering the *dre*-site-binding affinity of NagR by a factor of 1000 or 10 000 suffices to alleviate the NagR-mediated transcription repression. Thus, what at first seems puzzling, namely that the ligand GlcNAc-6-P binds to NagR with only millimolar affinity, would actually be sufficient to release the necessary amount of free energy that differentiates the specific from the non-specific binding mode. Key to the induction mechanism might therefore be the repositioning of the two DBDs in dimeric NagR in such a way that they cannot anymore bind simultaneously to the same *dre*-site. At the same time there seems to be no need to prevent binding of two DBDs from two distinct NagR dimers since this only results in nanomolar binding affinity (Figure 10).

As mentioned before, it cannot be excluded that a yet unknown and more potent effector might exist for NagR. It is also difficult to predict the relative amounts of DNA-bound, effector-bound and ligand-free NagR molecules in the actual cellular context. However, the results reported here suggest that an effector-induced extraordinary large conformational change in combination with a lowering of the DNA-binding affinity of NagR by a factor of 1000 might comprehensibly characterize the mechanism by which NagR regulates gene transcription.

## ACCESSION NUMBERS

The coordinates for the structures of NagR in complex with GlcN-6-P (PDB ID: 4U0V) and GlcNAc-6-P (PDB ID: 4U0W), as well as for the structures of full-length NagR in complex with 19mer palindromic dsDNA (PDB ID: 4WWC) and NagR–DBD in complex with 15mer palindromic dsDNA (PDB ID: 4U0Y) were deposited with the protein data bank.

## SUPPLEMENTARY DATA

Supplementary Data are available at NAR Online.

SUPPLEMENTARY DATA
