# Supplementary material for: Structural insight into operator dre-sites recognition and effector binding in the GntR/HutC transcription regulator NagR
Source: Nucleic Acids Res. 2015 Jan 6;43(2):1283–96. doi: 10.1093/nar/gku1374 (PMC4333415; doi:10.1093/nar/gku1374)
Supplement: SUPPLEMENTARY DATA [file supp_43_2_1283__index.html]

Structural insight into operator dre-sites recognition and effector binding in the GntR/HutC transcription regulator NagR — SUPPLEMENTARY DATA 

# Structural insight into operator *dre*-sites recognition and effector binding in the GntR/HutC transcription regulator NagR

## SUPPLEMENTARY DATA

**Files in this Data Supplement:**

- SUPPLEMENTARY DATA
