# Supplementary material for: Structural insight into operator dre-sites recognition and effector binding in the GntR/HutC transcription regulator NagR
Source: Nucleic Acids Res. 2015 Jan 6;43(2):1283–96. doi: 10.1093/nar/gku1374 (PMC4333415; doi:10.1093/nar/gku1374)
Supplement: SUPPLEMENTARY DATA [file supp_gku1374_nar-01946-m-2014-File012.pdf]

SUPPLEMENTARY EXPERIMENTAL PROCEDURES

Electrophoretic mobility shift assays

Samples for electrophoretic mobility shift assays (EMSA) were prepared in 20 mM Tris-HCl pH 7.5, 150 mM NaCl, mixed with 6x gel-loading dye (20 mM Tris-HCl pH 7.0, 30 % Glycerol), loaded on a 6 % (w/v) native polyacrylamide gel and run in TAE buffer (40 mM Tris, 20 mM acetic acid, 1 mM EDTA, pH 8.4) at 60 V. Gels were post-stained with a 3x GelRed^{TM} Nucleic Acid Stain solution (Biotium, Hayward, CA, USA) and analyzed *via* a UV-Transilluminator (Intas Science Imaging Instruments, Göttingen, Germany) to detect DNA-specific signals. Additionally, the gels were stained with a Coomassie Brilliant Blue solution to observe protein-specific signals (not shown), which in combination with the DNA-specific signals helped to identify protein-DNA complexes. Concentrations of the dsDNA constructs were kept constant at 2 μM, while protein concentrations varied from 0 μM to 12 μM.

SUPPLEMENTARY TABLES

Supplementary Table S1. Sequences of synthesized dsDNA fragments used in binding studies. The 14bp-long core regions involved in DBD binding are highlighted in grey. Nucleotides that disrupt the palindrome nature of the native sequences are shown in bold letters.

Construct	dsDNA constructs	Application
15mer palindromic dsDNA	5′-GTGGTCTAGACCACT-3′<br>3′-TCACCAGATCTGGTG-5′	EMSA / SEC / Crystallization
19mer palindromic dsDNA	5′-CAGTGGTCTAGACCACTGG-3′<br>3′-GGTCACCAGATCTGGTGAC-5′	Crystallization
15mer native *nagAB* dsDNA	5′-**C**TGGTCTAGA**T**CACT-3′<br>3′-T**G**ACCAGATCT**A**GTG-5′	EMSA / SEC
15mer native *nagP* dsDNA	5′-**T**TGGTATAGA**T**CACT-3′<br>3′-T**A**ACCATATCT**A**GTG-5′	EMSA
15mer palindromic dsDNA (shifted motif)	5′-GACCACATGTGGTCT-3′<br>3′-TCTGGTGTACACCAG-5′	EMSA
26mer palindromic dsDNA	Biotin-5′-GCTCAGGTGGTCTAGACCACTAGTCT-3′<br>3′-CGAGTCCACCAGATCTGGTGATCAGA-5′	SPR
26mer native *nagAB* dsDNA	Biotin-5′-GCTCAG**C**TGGTCTAGA**T**CACTAGTCT-3′<br>3′-CGAGTC**G**ACCAGATCT**A**GTGATCAGA-5′	SPR
26mer control dsDNA (derived from *nagA* gene)	Biotin-5′-GCAGAGAGTCTTCTTATTAAAGACAT-3′<br>3′-CGTCTCTCAGAAGAATAATTTCTGTA-5′	SPR
26mer control dsDNA (random design)	Biotin-5′-AATCATTTATGGCATAGGCAACAAGT-3′<br>3′-TTCGTAAATACCGTATCCGTTGTTCA-5′	SPR

Supplementary Table S2. Transcription factor-binding sites for NagR in *Bacillales*. Data were extracted from the RegPrecise database for transcription factor regulons (S1). Listed are the gene locus tag from the respective genome-sequencing project, the name of the respective gene, the position of the site with regard to the position of the first regulated gene and the sequence of the site. In the sequence the four-nucleotide-long motif of each half-site of the base-specific protein-DNA interaction is marked by a black box. The nucleotides that are predicted to interact with Gly69 (wing motif), Arg38 (α_D2) or Arg48 (α_D3) in NagR (*B. subtilis*) are colored in red.

Locus Tag	Name	Position	Sequence
Bacillus subtilis subsp. subtilis str. 168			
BSU07700	nagP	-68	AAATTGGTATAGATCACTAG
BSU35010	nagA	-72	CAGCTGGTCTAGATCACTAG
Bacillus amyloliquefaciens FZB42			
RBAM_032200	nagA	-73	TAACTGGTCTAGATAACTAG
RBAM_007900	nagP	-66	AAATTGGTATAGATCACTAG
Bacillus pumilus SAFR-032			
BPUM_3138	nagA	-71	TTACTGGTATAGACAACTAT
BPUM_0720	nagP	-87	AAATTGGTATAGATAACTAG
Bacillus licheniformis DSM 13			
BLi04348	nagA	-72	TTATTGGTCTAGACAACTAG
Geobacillus kaustophilus HTA426			
GK2277	nagA	-63	TTAGTGGTATAGACAACTAG
Bacillus cereus ATCC 14579			
BC4055	nagA	-99	TAGAATGTATAGACAACTAC
Bacillus halodurans C-125			
BH0421	nagA	-157	AAATTGGTATATACAAATTA
BH0422	nagP	-94	TAATAGGTATAGACAACTAA
BH0419	nagR	-153	TAATTGGTATAGACATTTTA
Bacillus clausii KSM-K16			
ABC1489	nagA	-79	TAATTGGTATAGACAACTAA
Oceanobacillus iheyensis HTE831			
OB0611	nagB	-55	TAACATGTATAGACAACTGT

SUPPLEMENTARY REFERENCES

S1. Novichkov, P.S., Laikova, O.N., Novichkova, E.S., Gelfand, M.S., Arkin, A.P., Dubchak, I. and Rodionov, D.A. (2010) RegPrecise: a database of curated genomic inferences of transcriptional regulatory interactions in prokaryotes. *Nucleic acids research*, **38**, 111-118.

S2. Sievers, F., Wilm, A., Dineen, D., Gibson, T.J., Karplus, K., Li, W., Lopez, R., McWilliam, H., Remmert, M., Soding, J. *et al.* (2011) Fast, scalable generation of high-quality protein multiple sequence alignments using Clustal Omega. *Molecular systems biology*, **7**, 539.

S3. UniProt, C. (2013) Update on activities at the Universal Protein Resource (UniProt) in 2013. *Nucleic acids research*, **41**, 43-47.

SUPPLEMENTARY FIGURES

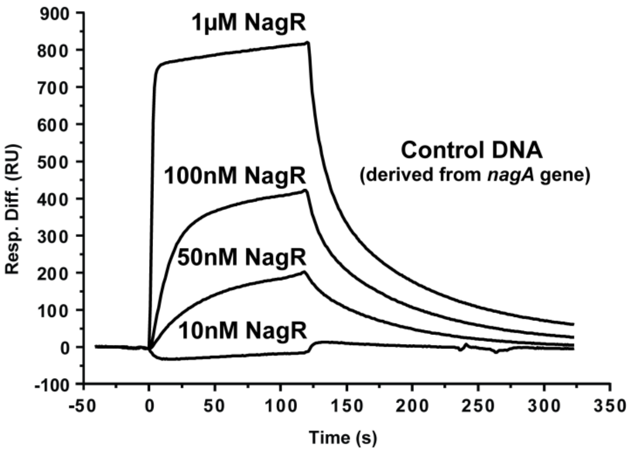

Supplementary Figure S1. SPR sensorgrams of the interaction between immobilized 26mer non-*dre*-site-containing control dsDNA (derived from *nagA* gene) and NagR at the indicated concentrations.

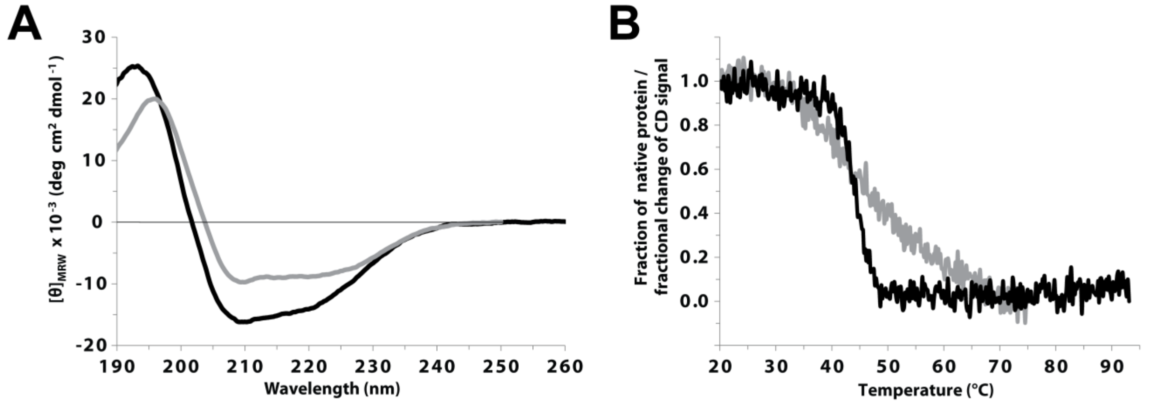

Supplementary Figure S2. Circular dichroism spectra of NagR and NagR-DBD.(**A**) Far UV circular dichroism spectra of NagR (black) and NagR-DBD (grey) recorded between 190 and 260 nm. (**B**) Thermal denaturation of NagR (black) and NagR-DBD (grey) monitored while increasing the temperature stepwise and recording the ellipticity at 208 and 200 nm wavelength, respectively. NagR-DBD (T_m = 39.8 ± 0.5 °C) displays a similar thermal stability than full-length NagR (T_m = 44.4 ± 0.1 °C).

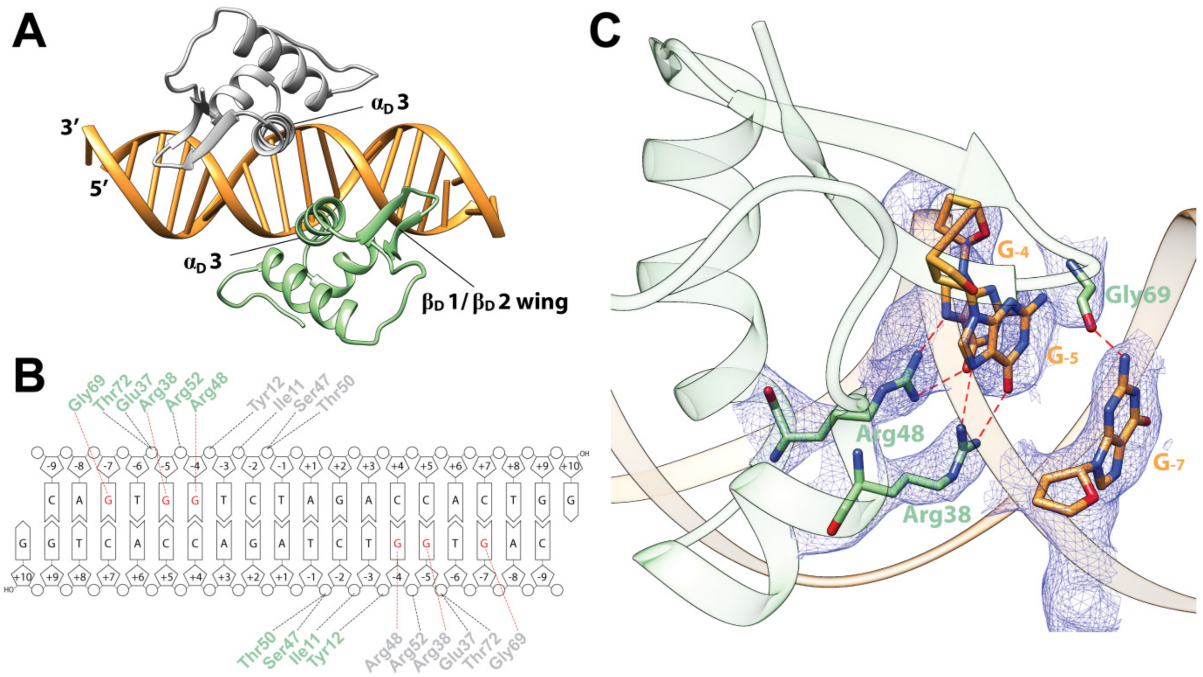

Supplementary Figure S3. Detailed view of the protein-DNA interactions in the complex of full-length NagR with palindromic dsDNA. (**A**) Close-up view of the NagR-DBD dimer bound to the 19mer palindromic dsDNA. Molecules are shown in a cartoon representation with the monomers (limited to residues 1 to 75) colored in light green and light grey. (**B**) Schematic summary of the contacts with DNA formed by the DNA-binding domains. Only direct interactions, identified with the analysis software NUCPLOT, are shown. Base-specific contacts are indicated in red. Nucleotides in the recognition half-sites are numbered according to their position from the center of the palindrome. (**C**) Details of the interactions between DNA and the DBD from chain B. Only base-directed interactions are shown. Hydrogen bonds are represented by red dotted lines. Interacting residues and bases are given as stick models and the 2F_o-F_c electron density map contoured as blue mesh at 1.0 σ is depicted within a radius of 2 Å around these residues and bases to illustrate the clear localization of the base-specific contacts.

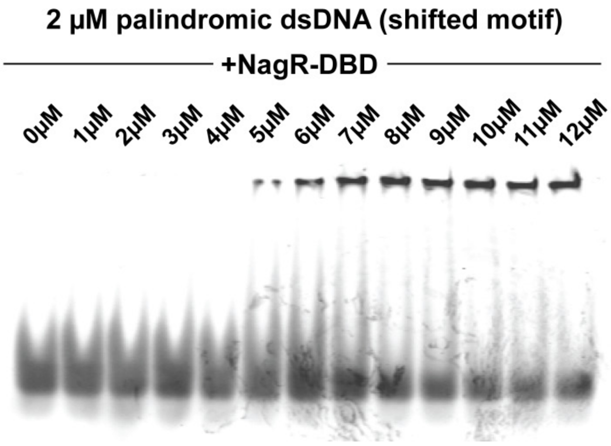

Supplementary Figure S4. Electrophoretic mobility shift assay showing the interaction of NagR-DBD with a palindromic 15mer dsDNA construct in which the GTGG-binding motif was shifted towards the 3'-end in both strands (Supplementary Table S1). Binding of NagR-DBD to this construct leads to a bridging of the dsDNA fragments and the formation of higher molecular weight oligomers. The final concentration of monomeric NagR-DBD is indicated for each lane. The dsDNA construct was used with a final concentration of 2 µM.

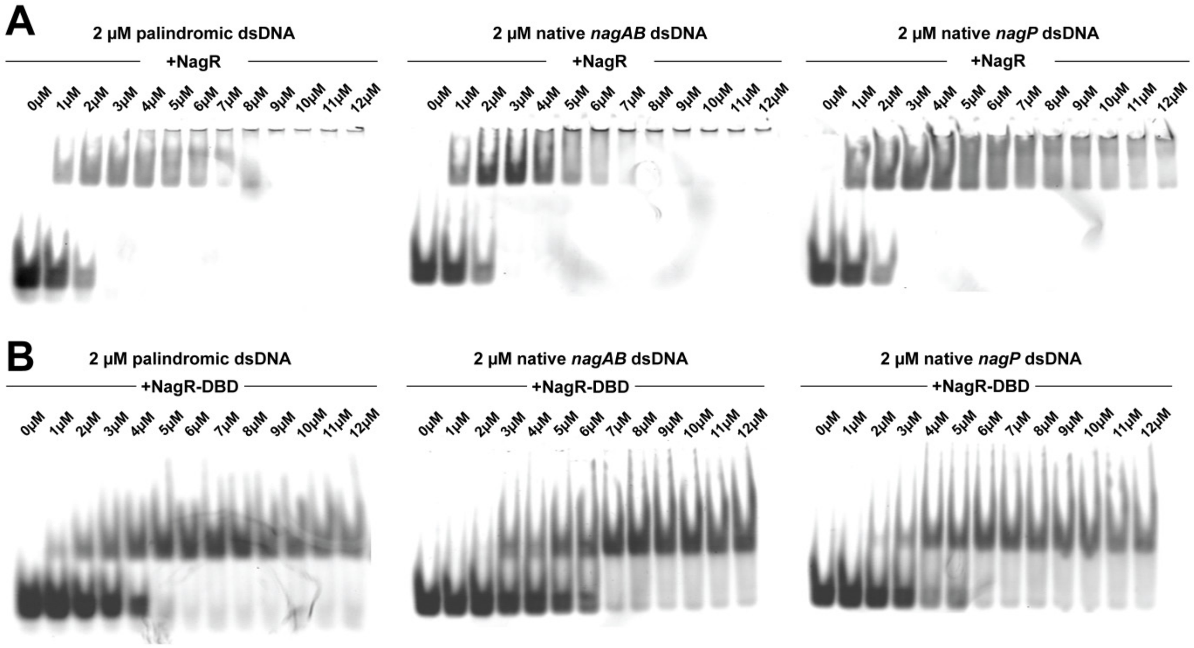

Supplementary Figure S5. Electrophoretic mobility shift assays showing the interaction of (**A**) NagR and (**B**) NagR-DBD with various 15mer dsDNA constructs derived from *Bacillus subtilis dre*-sites (Supplementary Table S1). The final concentrations of dimeric NagR or monomeric NagR-DBD, respectively, are indicated for each lane. The dsDNA constructs were used with a final concentration of 2 µM.

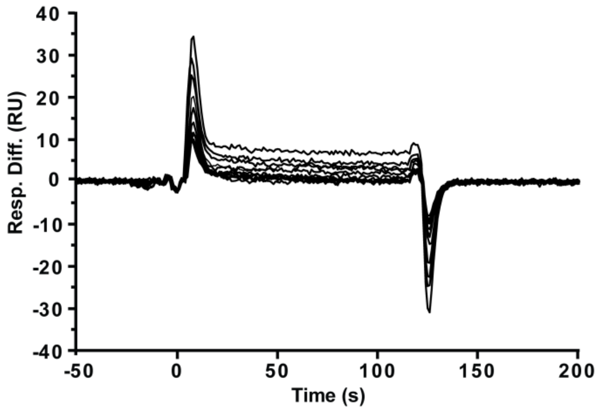

Supplementary Figure S6. No binding is observed when analyzing the interaction between NagR-DBD and non-*dre*-site containing dsDNA. Sensorgrams from SPR analyses of the interaction of NagR-DBD with randomly designed control dsDNA. The concentrations of NagR-DBD ranged from 50 nM to 10 µM. Signal variations most likely arise from bulk effects at high protein concentrations.

```
                                         αD1                    αD2
                                    hhhhhhhhhhhhhh            hhhhhhhh   h
NagR (B.s.)   ------------MNINKQSPIPIYYQIMEQLKTQIKNGELQPDMPLPSEREYAEQFGISR   48
DasR (S.c.)   MSTDVSSAENEGGATVRTARVPKYYRLKKHLLDMT--RTQTPGTPVPPERTLAAEFDTSR   58
                              : : :* **:: ::*          *  *:* **  * :*  **

                   αD3         βD1    βD2    β*/α*       αE1          βE1
              hhhhhhhhhhhh   ssss   ssss    ****      hhhhhhh    sssssssss
NagR (B.s.)   MTVRQALSNLVNEGLLYRLKGRGTFVSKPKMEQALQGLTSFTEDMKSRGMTPGSRLIDYQ   108
DasR (S.c.)   TTVRQALQELVVEGRLERIQGKGTFVAKPKVSQALQ-LTSYTEDMRAQGLEPTSQLLDIG   117
               ******.:** ** * *::*:****:***:.**** ***:****:::*: * *:*:*

                     αE2            βE2          βE3    αE3     αE4
              sss  hhhhhhh      sssssssssss  ssssssssssshhh     hhhh    hh
NagR (B.s.)   LIDSTEELAAILGCGHPSSIHKITRVRLANDIPMAIESSHIPFELAGELNESHF-QSSIY   167
DasR (S.c.)   YITADDRLAGLLDITAGGRVLRIERLRMANGEPMAIETTHLSAKRFPALRRSLVKYTSLY   177
               * : :.**.:*     . : :* *:*:**  *****::*:  :    *..* .  :*:*

                αE5          βE4        αE6            βE5           βE6
              hhhhhh    ssssssssssss  hhhhhhh      sssssssssss    ssssssss
NagR (B.s.)   DHIERYNSIPISRAKQELEPSAATTEEANILGIQKGAPVLLIKRTTYLQNGTAFEHAKSV   227
DasR (S.c.)   TALAEVYDVHLAEAEETIETSLATPREAGLLGTDVGLPMLMLSRHSQDRTGQPVEWVRSV   237
                : .  .: ::.*:: :* * ** .** :** : * *:*::.* :  :.*  .* .:**

                      βE7
                    sssssss
NagR (B.s.)   YRGDRYTFVHYMDRLS-         243
DasR (S.c.)   YRGDRYKFVARLKRPQD         254
              ******.**  :.* .
```

Residues of NagR involved in
DNA-binding | effector-binding

Supplementary Figure S7. Sequence alignment of NagR from *B. subtilis* and DasR from *S. coelicolor*. The sequence alignment was performed with Clustal Omega (S2) using the canonical protein sequences of entries O34817 and Q9K492 from the UniProt database (S3). Secondary structure elements refer to the topology of NagR and are marked with (h) or (s) for α-helices and β-strands, respectively. For a detailed classification, the familiar nomenclature $\alpha_{D/E}$ and $\beta_{D/E}$ is used. Residues of NagR involved in DNA- or effector-binding (as identified with NUCPLOT (29) and LigPlot+ (30)) are provided by a colored background as described in the figure. If these amino acids are fully conserved in DasR, they are additionally displayed in bold red letters. In general, fully conserved residues in both sequences are marked with an asterisk (*), while the conservation between groups of strongly and weakly similar properties is labeled with a colon (:) and a period (.), respectively.

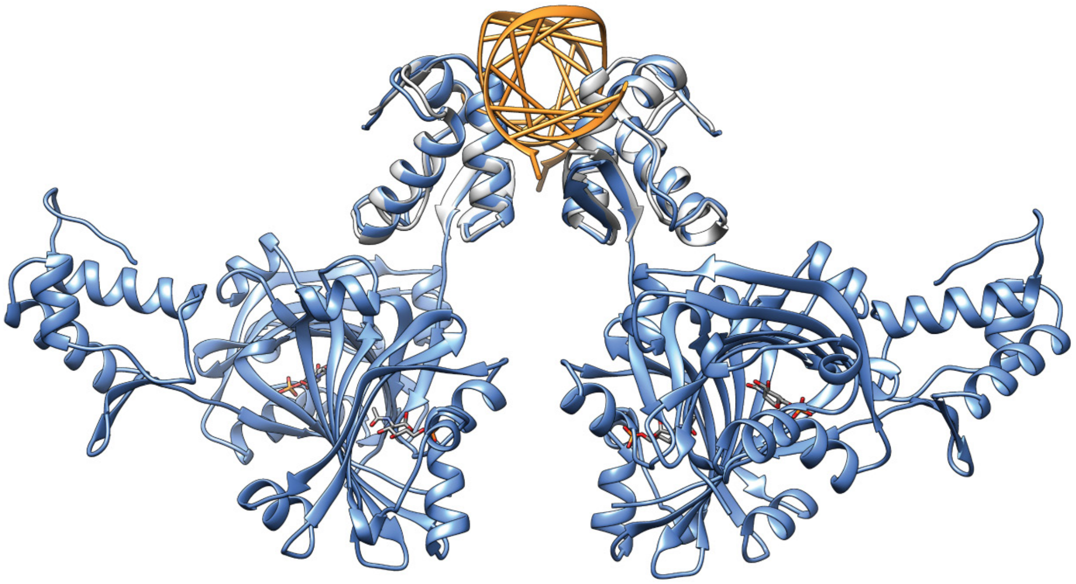

Supplementary Figure S8. Model for the *dre*-site-specific binding of effector-bound NagR to DNA. The model is derived from a superposition of the crystal structures of GlcNAc-6-P-bound NagR (shown in blue) and DNA-bound NagR-DBD (shown in grey).
